# Recent advances in the management of Hodgkin lymphoma

**DOI:** 10.12688/f1000research.8301.1

**Published:** 2016-04-27

**Authors:** Jose C. Villasboas, Stephen M. Ansell

**Affiliations:** 1 Department of Medicine, Division of Hematology, Mayo Clinic, Rochester, MN, USA

**Keywords:** Hodgkin lymphoma, cancer, lymph nodes, b lymphocytes

## Abstract

Hodgkin lymphoma (HL) is a rare cancer of the immune system that typically affects lymph nodes and sometimes other organs. Although the majority of patients can be potentially cured with the use of multi-agent chemotherapy and radiotherapy, a proportion of them will relapse or develop resistant disease for which treatment options are limited. In recent years, new agents have been developed and tested in HL with encouraging results. Two classes of drugs stand out as highly active in advanced HL based on recent study results: antibody-drug conjugates and programmed death 1 inhibitors. Clinical trials in HL with these agents have been completed in the past several years and the results have recently become available. In this review, we discuss the recent advances in the management of HL with a focus on strategies to decrease toxicity and a review of the two drug classes that have the potential to change the landscape of treatment of this disease.

## Introduction

Hodgkin lymphoma (HL) is a rare cancer that arises from immune cells known as B lymphocytes (B cells) and typically affects the lymph nodes and sometimes other organs ^1^. HL accounts for 10% of all lymphomas and less than 1% of all cancers diagnosed in the United States (US) yearly ^2^. It is a cancer of young adults, primarily occurring during the first four decades of life with a secondary peak between the sixth and seventh decades ^3^. Approximately 8500 new patients will be diagnosed with HL and 1120 will die of the disease in the US in 2016 according to projections ^2^.

HL is a curable cancer and current treatments can eradicate the disease in up to 80% of cases ^4^. Multi-agent chemotherapy, often in combination with radiation therapy, is the mainstay of management of HL, and treatment intensity is tailored to the risk of relapse. Despite the use of best available therapies, some patients will develop relapsed or refractory HL for which effective treatment options are limited. To meet the needs of these patients, new therapies are being tested in patients with HL and results are encouraging. These include agents that deliver cytotoxic chemotherapy to the interior of cancer cells using specific targets on the cell surface (antibody-drug conjugates [ADCs]) and strategies that enhance the ability of the patient’s immune system to eliminate HL cells (checkpoint inhibitors).

Here we provide an overview of the latest advances in the management of HL with a focus on the two classes of drugs that have gained the most visibility in recent years: ADCs and programmed death 1 (PD-1) receptor inhibitors.

## Antibody-drug conjugates

The term ADC describes a therapeutic agent designed to selectively deliver toxic compounds to the interior of cancer cells using a monoclonal antibody that recognizes a specific target. These agents aim to selectively target the malignant cells using the specificity of antibodies while minimizing collateral damage to normal tissue. This technology has now been tested in different cancers and there are currently two ADCs on the market in the US: trastuzumab emtansine and brentuximab vedotin (BV).

BV consists of a chimeric monoclonal antibody against human CD30 (cAC10) coupled to monomethyl auristatin E (MMAE) using a peptide linker. BV recognizes CD30 on the surface of the malignant HL cells and is internalized, releasing MMAE in its interior. Once inside the HL cell, MMAE prevents the polymerization of tubulin, a protein that is essential for cell division. Since CD30 is highly expressed on the surface of HL cells but not on most normal human tissue, BV can selectively target malignant cells to achieve its therapeutic effect.

Early studies of BV demonstrated encouraging activity in pre-clinical models ^5– 7^ and the drug was taken forward into initial clinical trials in patients with HL and anaplastic large cell lymphoma.

### Clinical activity of brentuximab vedotin in Hodgkin lymphoma

An initial phase I dose-escalation study investigated the safety and clinical activity of BV in 45 patients with relapsed CD30-positive lymphomas, 42 of them with HL ^8^. A total of 15 patients (36%) with HL achieved an objective response to treatment with BV, nine (21%) of whom had a complete response. Overall, the drug was well tolerated and safe at the dose level of 1.8 mg/kg given every 3 weeks, which moved forward into clinical development.

A multinational phase II study was then designed to evaluate the efficacy of BV in patients with advanced HL who had failed autologous stem cell transplantation (auto-SCT). A total of 105 patients with HL were treated with 1.8 mg/kg of BV every 3 weeks for a maximum of 16 cycles and assessed primarily for objective response. An objective response was observed in 76 patients (75%), including 35 (34%) complete remissions (CRs). The median duration of response was 6.7 months for all responders and 20.5 months for those achieving CR. The drug was well tolerated and the most common adverse effects were peripheral neuropathy, fatigue, gastrointestinal symptoms, and neutropenia. There were no cases of febrile neutropenia or deaths attributed to BV.

Based on the results of this trial, BV received accelerated approval from the US Food and Drug Administration (FDA) on 19 August 2011 for the treatment of patients with HL after failure of auto-SCT or after failure of at least two prior multi-agent chemotherapy regimens in patients who are not candidates for auto-SCT. Updated results from that trial after a median follow-up of 3 years confirmed that responses were durable, especially for the group achieving CR ^9^.

Additional studies were designed to evaluate the role of BV in other settings. A large phase III trial demonstrated increased progression-free survival (PFS) when BV was used as consolidative treatment following auto-SCT ^10^. A phase I dose-escalation trial studied the use of BV + ABVD (doxorubicin, bleomycin, vinblastine, and dacarbazine) or BV + AVD (ABVD without bleomycin) in treatment-naïve patients with HL ^13^. Fifty-one patients were treated and CR was achieved in 95% of BV + ABVD and 96% of BV + AVD patients. Aside from excessive pulmonary toxicity in the BV + ABVD group, treatment was generally well tolerated. A large multicenter phase III trial of BV + AVD versus ABVD (NCT01712490) for newly diagnosed advanced HL has recently completed accrual.

### Toxicities associated with brentuximab vedotin

BV has been approved for use as a single agent at the dose of 1.8 mg/kg (up to 180 mg) administered intravenously every 3 weeks until a maximum of 16 cycles, disease progression, or unacceptable toxicity. At this dose level, BV is generally well tolerated. The most clinically relevant side effect of BV is peripheral neuropathy. Sensory neuropathy is more commonly seen (42%) than motor neuropathy (11%). Cases of BV-induced neuropathy may be severe (up to 31% at least grade 2) and constituted the leading cause of drug discontinuation in some of the clinical trials ^12, 13^. Median time to onset of neuropathy was about 12 weeks and severity was cumulative. The majority of patients (80%) experienced some improvement of symptoms (of at least one grade) with drug discontinuation but complete resolution was observed in only half of the cases. It is important to mention that the incidence of febrile neutropenia was extremely low, making BV an attractive drug for combination with multi-agent chemotherapy regimens.

The combination of BV with bleomycin in a phase I trial investigating the use of BV in combination with standard therapy (ABVD) led to unacceptable pulmonary toxicity ^11^. On the basis of these findings, the label of BV was modified to include a contraindication to its use in conjunction with bleomycin. Consequently, the trials currently evaluating the use of BV in combination with chemotherapy have modified the regimens to exclude bleomycin.

Although BV is generally safe and well tolerated, two other uncommon but serious adverse events potentially associated with the use of BV are worth mentioning: progressive multifocal leukoencephalopathy (PML) ^14, 15^ and pancreatitis ^16^. Post-marketing reports linked BV to these serious and potentially life-threatening conditions. These resulted in the addition of a black-box warning for the risk of PML and raised awareness for the occurrence of acute pancreatitis in patients treated with BV.

### Other antibody-based conjugates currently in development for Hodgkin lymphoma

The success of BV – the first ADC approved for the treatment of lymphomas – has spurred interest in developing new agents using the same platform. ADCT-301 is an ADC that combines a monoclonal antibody against CD25 to an antibiotic with anti-tumoral properties (pyrrolobenzodiazepine or PBD). This compound has demonstrated pre-clinical activity ^17^ and is now accruing patients with HL for a phase I study (NCT02432235). Another compound targeting CD25 combines the specificity of a monoclonal antibody (daclizumab) to the anti-tumoral activity of radiotherapy (RT) by linkage to a beta-emitter particle ( ^90^Y). ^90^Y-daclizumab has now been tested in patients with advanced HL in a recently published phase II study with encouraging results ^18^.

## Checkpoint inhibitors

The normal immune system is constantly monitoring the body for infections and cancer cells. Once activated, immune cells are subject to regulatory checkpoints designed to extinguish the immunological response once the offender has been eliminated. These natural regulatory mechanisms are meant to prevent uncontrolled immune activation, which could lead to untoward damage to normal tissue. These pathways are collectively known as immune checkpoints, and our knowledge of these mechanisms is rapidly increasing. Cancer cells, however, can highjack immune checkpoint pathways to actively evade immune surveillance.

One such example is the overexpression of ligands for the PD-1 receptor on the surface of cancer cells. Once these ligands (PD-L1 or PD-L2) engage the PD-1 receptor expressed on the surface of activated T cells, they lead to a cascade of events that culminate in decreased function and survival of immune cells. The ultimate effect is dampening of the immune response, which allows the tumor to progress unopposed. The use of this evasion mechanism by HL cells has been clearly described ^19– 22^. Drugs that interfere with the interaction between PD-1 and its ligands (PD-L1 or PD-L2) have been developed and tested in different cancer types.

Two of these PD-1 inhibitors – nivolumab and pembrolizumab – have now been tested in patients with advanced HL with remarkable results, which will be discussed here.

### Clinical activity of nivolumab in Hodgkin lymphoma

Nivolumab is a monoclonal antibody that binds to the PD-1 receptor and prevents it from interacting with its ligands (PD-L1 and PD-L2). By disrupting the PD-1:PD-L1/2 axis, nivolumab seeks to overcome immune tolerance induced by cancer cells, thereby releasing the immune system for an effective anti-cancer response. Nivolumab has now been tested in several malignancies and received FDA approval for use in melanoma, non-small cell lung cancer, and renal cell carcinoma ^23^.

A phase I dose-escalation trial tested the safety and efficacy of nivolumab in patients with heavily pre-treated advanced HL. The original manuscript reported on the experience with the first 23 patients treated at the 3 mg/kg dose level after a median follow-up of 40 weeks ^24^. An objective response was observed in 20 (87%) patients, including four (17%) with a complete response. The additional three patients had stable disease, indicating that all 23 patients derived some degree of clinical benefit from the treatment. Nivolumab was well tolerated and the safety profile was consistent with previous experience documented for other tumor types. No serious adverse events or deaths attributed to the drug were observed.

Updated long-term results on these patients after a median follow-up of 86 weeks were presented at the most recent American Society of Hematology (ASH) annual meeting ^25^. Of the 20 initial responders, 10 demonstrated responses lasting over 41 weeks (range 41.7 to 90.7 weeks), including one who was retreated at progression after treatment was discontinued. Of the remaining 10 responders, four developed progressive disease, one discontinued due to adverse effects (without progression), and five discontinued nivolumab to undergo allogeneic SCT. These results provide early evidence that responses obtained with nivolumab in HL may be durable.

Based on these encouraging early results, nivolumab received breakthrough therapy designation for HL from the FDA. A registrational phase II trial utilizing nivolumab for the treatment of HL is currently underway (NCT02181738).

### Pembrolizumab

Pembrolizumab is another monoclonal antibody that belongs to the class of PD-1 inhibitors. Similar to nivolumab, this agent works by targeting the PD-1 receptor and is approved by the FDA for the treatment of melanoma and non-small cell lung cancer ^26^.

The safety and clinical activity of pembrolizumab is being tested in a phase Ib multicenter clinical trial for patients with relapsed/refractory HL after failure of auto-SCT and BV. Patients who were deemed ineligible for or refused auto-SCT were also included. Patients were treated with 10 mg/kg of pembrolizumab every 2 weeks. An updated report on the first 31 patients was presented at the most recent ASH annual meeting ^27^. At a median follow-up of 9.7 months, an objective response was observed in 20 (65%) patients including five (16%) achieving CR. An additional seven (23%) patients had stable disease as best response, indicating that 87% of patients derived clinical benefit. Pembrolizumab was well tolerated and toxicity was consistent with previous experience in other cancers. Fourteen of the 20 responses were ongoing at the time of data cut-off. No serious adverse events or deaths were attributed to the treatment.

These results reinforced the notion that PD-1 inhibition is a safe strategy with a strong signal of clinical activity in heavily pre-treated HL patients. These and other studies are currently open and accruing patients with HL.

### Adverse effect profile of programmed death 1 inhibitors

PD-1 inhibitors are usually well-tolerated drugs, even for patients who are heavily pre-treated with standard cytotoxic agents. The most common adverse effects of this class are dermatologic (rash and pruritus), metabolic (lipid changes, hyperglycemia, hypoalbuminemia, and electrolyte imbalances), hematologic (anemia and lymphopenia), gastrointestinal (changes in bowel habits and nausea/vomiting), and respiratory (cough and dyspnea) ^26, 28^ in nature and also include fatigue, abnormal liver enzymes, and arthralgia.

A distinct class of adverse effects – collectively known as immune-related adverse effects (IrAEs) – deserves special mention, since they are uniquely associated with these immunotherapeutic agents ^29^. These are the result of the immune-stimulatory effects of these drugs and can affect different organs with pleomorphic presentations. Despite being uncommon, early recognition and management is crucial, as these events can progress rapidly to cause significant morbidity or even death. Potentially affected organs are the lungs (pneumonitis), the endocrine system (hypophysitis, thyroiditis, and adrenal insufficiency), the skin (toxic epidermal necrolysis), and the gastrointestinal tract (colitis and pancreatitis). These occurrences should trigger immediate evaluation and providers should have a low threshold to hold therapy (for mild cases) and treat with systemic steroids (for moderate to severe cases). Expert consultation with organ-specific specialties is highly advised, as these patients often need invasive testing (i.e. bronchoscopies and colonoscopies) to rule out alternative diagnoses (i.e. infection or tumor progression). Standardized guidelines on the management of these patients are not yet available and treatment must be individualized.

Caution is advised when utilizing checkpoint inhibitors in patients with a documented history of autoimmune disorders, especially if poorly controlled. The same applies for the use of checkpoint inhibitor therapy in patients who have failed allogeneic SCT and have active graft-versus-host disease (GVHD). Evidence is starting to surface supporting the safety of the use of these agents in patients with pre-existing autoimmune conditions ^30^ (as long as they are adequately controlled) or after allogeneic transplant (as long as GVHD is minimal) ^31– 34^. These are mostly retrospective studies or small case series; therefore, therapy in these special situations must be individualized and include a thorough discussion of the risks, benefits, and uncertainties.

## Other immunotherapeutic strategies in development for Hodgkin lymphoma

The exciting responses observed with the use of PD-1 inhibitors in HL resulted in the development of a number of strategies combining these drugs with other agents in an attempt to increase efficacy. Clinical trials combining PD-1 inhibitors to Bruton tyrosine kinase inhibitors (NCT02362035), bispecific NK-cell engager antibodies (NCT02665650), BV (NCT01896999 and NCT02572167), other immune checkpoint inhibitors (NCT01896999, NCT02304458, and NCT01592370) or standard cytotoxic chemotherapy (NCT02181738) are under active development.

Another strategy to manipulate the immune system against the cancer cell is the use of chimeric antigen receptor-modified T cell (CAR-T) therapy. This involves genetically re-engineering the patient’s effector immune cells (T cells) to directly recognize and eliminate tumor cells via modifications to the T cell receptor ^35^. Exciting results were observed when this technology was tested in a pivotal study of patients with relapsed acute lymphoblastic leukemia ^36^. The principle has now been applied to target CD30 – expressed on the surface of HL cells – and preclinical models demonstrate encouraging activity ^37^. At least seven studies evaluating the use of CAR-T therapy in HL are currently open.

## Response-adapted strategies for early stage Hodgkin lymphoma

Patients with stages I/II HL have a chance of cure above 90% when treated with a combination of multi-agent chemotherapy and RT. Unfortunately, long-term side effects of treatment are still a reality that may negatively impact the quality of life and morbidity of long-term survivors. Recent strategies have focused on de-escalating treatment in lower risk patients based on an interim assessment using positron-emission tomography (PET) scans. These response-adapted strategies aim to identify patients in whom therapy may be safely de-escalated to minimize long-term toxicity without compromising efficacy.

Two studies have recently reported the use of interim PET scans in response-adapted therapy for early stage HL. The Randomised Phase III Trial to Determine the Role of FDG–PET Imaging in Clinical Stages IA/IIA Hodgkin’s Disease (RAPID) randomized 426 patients with a negative PET after three cycles of ABVD to receive either RT or no further treatment using a non-inferiority design ^38^. The PFS at 3 years was 94.6% in the RT group and 90.8% in the observation group. Although the trial failed to demonstrate that this strategy was non-inferior to RT, the results highlighted the low incidence of recurrence in early stage patients who are able to achieve a negative PET scan following initial chemotherapy.

A second study from the US Intergroup had interim results presented at the most recent ASH annual meeting ^39^. In this phase II study, patients with stages I/II HL were assessed with a PET scan after two cycles of ABVD. Patients with a positive PET went on to receive escalated therapy with two cycles of dose-intense bleomycin, etoposide, doxorubicin, cyclophosphamide, vincristine, procarbazine, and prednisone (escalated BEACOPP) + RT. Patients with a negative PET received only two additional cycles of ABVD. At a median follow-up of 2 years, PFS was 92% in PET-negative patients compared to 66% in the PET-positive group. These results again suggested excellent outcomes with an abbreviated chemotherapy-only approach.

Together, these results indicate that an interim PET scan is a powerful biomarker with predictive value in patients with early stage HL. Its use has now been incorporated into clinical practice guidelines for a select group of patients ^40^.

## Future perspectives in the management of Hodgkin lymphoma

In an era when immunotherapy has emerged as the next frontier in cancer management, HL consistently stands out as a successful platform for the development of these strategies. One must not forget, however, the tremendous success that has been achieved up to this point by virtue of treatment with standard cytotoxic agents and RT. Unfortunately, negative long-term consequences of these therapies can be significant for HL survivors. This constitutes both the challenge and the opportunity that will need to be addressed by the next generation of clinical trials. The challenge consists of incorporating these highly active agents into well-established standard-of-care treatment paradigms without compromising efficacy for this highly curable disease. The opportunity that unfolds involves a potential for de-escalation of treatment intensity by incorporating these newer agents and metabolic biomarkers such as PET scans. Similarly, these new agents provide our elderly and frail patients with treatment opportunities that were not previously available. As long as this can be achieved safely while retaining efficacy, the potential to increase the cure rate and reduce long-term toxicity is significant.

## Abbreviations

ABVD, doxorubicin, bleomycin, vinblastine, and dacarbazine; ADC, antibody-drug conjugate; ASH, American Society of Hematology; auto-SCT, autologous stem cell transplantation; BV, brentuximab vedotin; CAR-T, chimeric antigen receptor-modified T cell; CR, complete remission; escalated BEACOPP, dose-intense bleomycin, etoposide, doxorubicin, cyclophosphamide, vincristine, procarbazine and prednisone; FDA, US Food and Drug Administration; GVHD, graft-versus-host disease; HL, Hodgkin lymphoma; IrAEs, immune-related adverse effects; MMAE, monomethyl auristatin E; PBD, pyrrolobenzodiazepine; PD-1, programmed death 1; PD-L1, programmed death receptor ligand 1; PD-L2, programmed death receptor ligand 2; PET, positron-emission tomography; PFS, progression-free survival; PML, progressive multifocal leukoencephalopathy; RT, radiotherapy; SCT, stem cell transplantation.
